# Use of a Real-Time Locating System to Assess Internal Medicine Resident Location and Movement in the Hospital

**DOI:** 10.1001/jamanetworkopen.2022.15885

**Published:** 2022-06-08

**Authors:** Michael A. Rosen, Amanda K. Bertram, Monica Tung, Sanjay V. Desai, Brian T. Garibaldi

**Affiliations:** 1 Armstrong Institute for Patient Safety and Quality, Johns Hopkins University School of Medicine, Baltimore, Maryland; 2 Division of General Internal Medicine, Johns Hopkins University School of Medicine, Baltimore, Maryland; 3 medical student at Johns Hopkins University School of Medicine, Baltimore, Maryland; 4 currently with Department of Medicine, University of California at San Francisco; 5 Division of Pulmonary and Critical Care Medicine, Johns Hopkins University School of Medicine, Baltimore, Maryland; 6 Chief Academic Officer, American Medical Association

## Abstract

**Importance:**

The patient-physician clinical encounter is the cornerstone of medical training, yet residents spend as little as 12% of their time in direct patient contact.

**Objectives:**

To use a real-time locating system (RTLS) to characterize intern work experiences in the hospital, understand factors associated with time spent at patients’ bedsides, and inform future interventions to increase time spent with patients.

**Design, Setting, and Participants:**

This cross-sectional study was conducted from July 1, 2018, to June 30, 2019 (ie, the academic year 2018-2019). Internal medicine residents from postgraduate year 1 (interns) at an academic medical center wore an infrared badge that recorded location and duration (eg, patient room, ward hall, physician workroom). Data were analyzed from September 1, 2020, to August 30, 2021.

**Main Outcomes and Measures:**

Main outcome was time (in minutes) at the bedside; the unit of analysis was a 24-hour intern day or interval of time within the day (eg, rounding period). Descriptive statistics are reported overall, by intern, and for 5 clinical service categories. Multilevel modeling assessed the association of intern, service, and calendar time with time spent at the bedside.

**Results:**

Data from 43 of 52 interns (82.7%) encompassing 95 275 hours of observations were included for analyses. Twenty-six interns (60.5%) were women. Interns were detected for a mean (SD) of 722.8 (194.4) minutes per 24-hour period; 13.4% of this time was spent in patient rooms (mean [SD] time, 96.8 [57.2] minutes) and 33.3% in physician workrooms (mean [SD] time, 240.9 [228.8] minutes). Mean percentage of time at the bedside during a 24-hour period varied among interns from 8.8% to 18.3%. Mean (SD) percentage of time at the bedside varied by service for the 24-hour period from 11.7% (6.6%) for nononcology subspecialties to 15.4% (6.0%) for oncology, and during rounds from 8.0% (12.4%) for nononcology subspecialties to 26.5% (12.1%) for oncology. In multilevel modeling, the individual intern accounted for 8.1% of overall variance in time spent at the bedside during a 24-hour period, and service accounted for 18.0% of variance during rounds.

**Conclusions and Relevance:**

The findings of this cross-sectional study support previous evidence suggesting that interns spend only a small proportion of time with hospitalized patients. The differences in time spent in patients’ rooms among interns and during rounds constitute an opportunity to design interventions that bring trainees back to the bedside.

## Key Points

**Question:**

Can a real-time locating system (RTLS) provide scalable information about where internal medicine residents spend their time in the hospital?

**Findings:**

In this cross-sectional study, 43 internal medicine interns wore an RTLS badge during the 2018-2019 academic year with usable data. Interns spent only 13.4% of their time in patient rooms; there was significant interindividual variation in bedside time and significant interservice differences in time spent in patient rooms during rounds, with most time spent in the hallway or the workroom.

**Meaning:**

These findings suggest that an RTLS can provide granular information about where medical residents spend their time in the hospital and can inform interventions to improve the training environment.

## Teaser

This cross-sectional study uses a real-time locating system to characterize work experiences and location in the hospital among internal medicine residents, particularly time spent at patients’ bedsides.

## Introduction

The patient-physician encounter remains the cornerstone of medical practice. However, medical residents spend as little as 12% of their time in direct patient care activities.^1,2,3,4,5,6^ Traditional rounds have increasingly migrated to hallways and conference rooms.^7^ These trends decrease the amount of time available to practice clinical skills and likely have contributed to an overall decline in skills.^8,9,10^ This decline matters because a large proportion of medical errors are linked to oversights during the physical examination.^11,12^ Limited time with patients may also lessen physicians’ sense of purpose, likely adding to a rise in burnout, particularly among trainees and junior faculty.^13,14,15,16^ We must reexamine the current training environment to jointly optimize clinical skills development and professional fulfillment.^17^

Time-motion studies are 1 option to capture resident workflows and inform work redesign efforts.^18^ However, they require trained observers shadowing participants and have logistical challenges and high costs. Methods that use a real-time locating system (RTLS)^19,20^ may be a more scalable approach for graduate medical training programs.^21^

This study used RTLS methods to characterize intern work experiences in the hospital, particularly time spent at the bedside. We aimed to understand factors associated with time at the bedside to inform future interventions that increase time spent with patients.

## Methods

### Study Design, Participants, and Setting

This cross-sectional study used an RTLS to track the physical location of internal medicine residents (referred to hereinafter as interns) from postgraduate year 1 at The Johns Hopkins Hospital, Baltimore, Maryland, from July 1, 2018, to June 30, 2019 (ie, the 2018-2019 academic year). All interns (N = 52) were eligible to participate and attended a 30-minute presentation during orientation, when they were given an RTLS badge (Midmark, Inc) attached to their identification badge lanyard. Interns were instructed to keep the badge attached to their lanyard while in the hospital. They could opt out of the study at any time by returning their badge to the program coordinator and could reenter the study by requesting another badge. This study was classified as nonhuman participant research and as quality improvement by the Johns Hopkins University School of Medicine Institutional Review Board; therefore, informed consent was not required. This report adheres to the Strengthening the Reporting of Observational Studies in Epidemiology (STROBE) guideline.

### Variables and Data Sources

The RTLS technology (Executone Systems) was piloted in January 2018 and found feasible for tracking resident time and location in a large academic medical center.^19^ Each RTLS badge has a unique identification number and emits an infrared pulse detected by environmental sensors (ie, receivers) built into the hospital structure. In most hospital units, all patient rooms, physician workrooms, nurses’ stations, and ward hallways have unique environmental sensors that can localize the badge wearer to a discrete physical location, at a temporal resolution of 3 seconds (ie, the rate of infrared pulses from the badge). We grouped 14 different clinical service rotations into 5 service categories: house staff (general medicine rotation led by chief residents), hospitalist (general medicine rotation led by hospitalists), intensive care unit (ICU; both medical and cardiac), oncology (a department outside the Department of Medicine), and nononcology subspecialties (cardiology, gastroenterology, renal transplant, and neurology). This study focused on inpatient rotations because RTLS receivers are not installed in outpatient clinics. A research coordinator (A.K.B.) kept a secure file with names and badge numbers for the purpose of linking RTLS data to rotation schedules. Data were analyzed by badge number only and reported in aggregate.

The RTLS data consisted of rows for participant detections in an epoch time series, with columns for badge number, receiver number, brief description of the receiver location, and time-in and time-out time stamps. Duration (in minutes) for each detection was calculated using the time stamps. Data were prepared for analysis in 2 steps by 1 of the authors (M.A.R.). First, receivers were categorized by location: ward hall, transit (elevators, stairways, halls between clinical units, main hospital hallways), patient room, staff area (eg, conference rooms, break rooms), physician workroom, supply (eg, medication, nourishment), family waiting space, education space, and other or unknown. Bathrooms, on-call rooms, and cafeterias do not have discrete RTLS receivers; most are captured under the transit category. Second, the data were cleaned, which involved setting time thresholds for unrealistically long durations for a location detection (>300 consecutive minutes in a physician workroom, ≥10 minutes in elevators and hallways connecting units, >180 minutes at any other location) and changing those locations to other or unknown. Unrealistic durations typically occurred if the badge was left in a workroom or an intern left an area without being detected elsewhere (eg, if the badge was covered up by a coat). These thresholds were determined by 2 authors (M.A.R. and B.T.G.) before analysis based on previous pilot RTLS data.^19^

### Statistical Analysis

Our analyses are primarily descriptive and report proportions and means (SDs). Data were analyzed from September 1, 2020, to August 30, 2021.

#### Overall Amount of Time at the Bedside

Overall amount and percentage of time spent in locations was summarized for 6 intervals: a 24-hour day, typical rounding hours (8:30 am to 11:00 am), morning (6:00 am to 12:00 pm), afternoon (12:00 pm to 6:00 pm), evening (6:00 pm to 12:00 am), and night (12:00 am to 6:00 am). Descriptive measures were generated for all days combined and for weekdays and weekends separately. To be included for analysis, each intern day required at least 4 hours of location data, because this was estimated to be the minimum shift duration when an intern would have inpatient clinical duties and interact with patients. The only exception was the 2.5-hour rounding period, in which at least 1 hour of location data was required.

#### Individual Differences Between Interns in Time at the Bedside

For each intern, we calculated the amount and percentage of time spent in patient rooms for each day and report the mean (SD) percentage of time at the bedside for a 24-hour period. A box plot is used to compare individual interns; the IQR is shown for all data. Overall amount of time at the bedside for the 1-year study period is reported.

#### Differences in Time and Changes Over Time at the Bedside

Descriptive statistics for overall time in patient rooms, ward halls, and physician workrooms by service and during rounds were calculated. We used 3 factors associated with outcomes to test trends over time: day of week (0-6), day of service rotation (0-13), and day of year (0-363) beginning July 1. We used multilevel modeling^22^ to address the above aims with R, version 4.02 (R Project for Statistical Computing),^23^ and the lme4 package, version 1.1.27.1 (R Project for Statistical Computing).^24^ Separate models were constructed for the overall 24-hour interval and rounding. A null model or unconditional means model of percentage of time at the bedside was created for each interval and served as a benchmark for further modeling. Intern was added as a random variable for model 1, service was added for model 2, and time-based factors associated with outcomes were added for model 3. Intraclass correlation coefficients measured the proportion of variance in percentage of time at the bedside between different interns and services. Model deviance was computed to compare model fit using an L ratio test, and α < .05 was used to assess significance, indicating a better fitting model than the null model. For example, if model 1 was a better-fitting model, this would indicate that intern is a valid grouping variable, demonstrating significant differences in time at the bedside between interns. Time at the bedside was grand mean centered and scaled by dividing the sample SD before multilevel modeling analysis.^22^ All statistical tests were 2 tailed and used α <.05 to indicate statistical significance.

## Results

Forty-four of 52 interns (84.6%) agreed to participate. Data for 1 badge were dropped owing to low volumes and irregularities (only 6 days of data recorded, and no time registered in patient rooms). After this exclusion, data from 43 interns (82.7%) encompassing 2 040 442 badge location detections and 95 275 hours of observations were included for analyses. Of these, 26 interns (60.5%) were women and 17 (39.5%) were men. Race and ethnicity data were not collected. A total of 9712 badge detections (0.5% of total detections) were unrealistically long and recategorized as other or unknown. These detections represented 8645.8 hours, or 9.1% of the total person-hours collected (additional details on recategorized detections are provided in eTables 1 and 2 in the Supplement).

### Overall Amount of Time at the Bedside

Interns were detected for a mean (SD) of 722.8 (194.4) minutes per 24-hour period; 13.4% of this time was spent in patient rooms (mean [SD] time, 96.8 [57.2] minutes), 23.7% in ward halls (mean [SD] time, 171.1 [142.3] minutes), and 33.3% in physician workrooms (mean [SD] time, 240.9 [228.8] minutes). Time in patient rooms varied by time of day, ranging from 11.6% during afternoons to 17.8% during evenings. Percentage of time at the bedside was similar across time intervals on weekdays and weekends (Table 1 and eFigure 1 in the Supplement). The largest difference was between weekday afternoons (10.9% of time at the bedside) and weekends (14.3% of time at the bedside for 24-hour and afternoon periods).

**Table 1.** Time in Locations by Time of Day and Day of Week^a^

Location category	Overall			Weekdays			Weekends		
	Person-days	Mean (SD) time, min	Time, %	Person-days	Mean (SD) time, min	Time, %	Person-days	Mean (SD) time, min	Time, %
12:01 am to 12:00 am (all day)									
Patient room	7909	96.8 (57.2)	13.4	6136	94.6 (55)	13.1	1773	104.5 (63.9)	14.3
Physician workroom	7909	240.9 (228.8)	33.3	6136	241.8 (225.1)	33.5	1773	237.7 (241.3)	32.6
Ward hall	7909	171.1 (142.3)	23.7	6136	171.1 (140.5)	23.7	1773	171.1 (148.5)	23.5
Other or unknown	7909	57.7 (109.8)	8.0	6136	56.3 (103.5)	7.8	1773	62.4 (129.1)	8.6
Staff area	7909	87.7 (141.0)	12.1	6136	83.8 (136.8)	11.6	1773	101.1 (153.7)	13.9
Transit^b^	7909	43.8 (37.7)	6.1	6136	43.9 (36.7)	6.1	1773	43.5 (41.0)	6.0
Education space	7909	15.5 (24.0)	2.1	6136	19.8 (25.6)	2.7	1773	0.3 (2.9)	0.04
Supply	7909	4.3 (11.0)	0.6	6136	4.3 (11.4)	0.6	1773	4.1 (9.6)	0.6
Family waiting space	7909	5.2 (16.4)	0.7	6136	5.5 (16.8)	0.8	1773	4.2 (15.0)	0.6
Total	7909	722.8 (194.4)	NA	6136	721.0 (190.1)	NA	1773	728.8 (208.6)	NA
8:30 am to 11:00 am (rounds)									
Patient room	13 226	20.6 (20.2)	16.0	10 624	20.8 (20.0)	16.3	2602	19.7 (20.7)	15.0
Physician workroom	13 226	32.5 (45.3)	25.3	10 624	31.6 (44.3)	24.7	2602	36.6 (49.0)	28.0
Ward hall	13 226	48.6 (40.0)	37.8	10 624	47.8 (39.3)	37.4	2602	51.6 (42.3)	39.4
Other or unknown	13 226	8.2 (20.0)	6.4	10 624	8.7 (19.9)	6.8	2602	5.8 (20.1)	4.4
Staff area	13 226	7.2 (15.6)	5.0	10 624	6.9 (15.3)	5.4	2602	8.8 (16.8)	6.7
Transit^b^	13 226	5.9 (9.2)	4.6	10 624	5.8 (8.7)	4.5	2602	6.5 (10.9)	5.0
Education space	13 226	3.1 (13.3)	2.4	10 624	3.8 (14.7)	3.0	2602	0.01 (0.2)	0.01
Supply	13 226	0.7 (4.0)	0.5	10 624	0.7 (4.1)	0.5	2602	0.7 (3.3)	0.5
Family waiting space	13 226	1.6 (7.5)	1.2	10 624	1.7 (7.9)	1.3	2602	1.2 (5.6)	0.9
Total	13 226	128.5 (21.6)	NA	10 624	127.9 (22.0)	NA	2602	130.9 (19.7)	NA
6:01 am to 12:00 pm (morning)									
Patient room	5865	50.0 (31.1)	14.9	4659	50.3 (30.7)	14.9	1206	49.1 (32.7)	15.0
Physician workroom	5865	101.5 (106.0)	30.2	4659	102.2 (104.4)	30.3	1206	98.7 (112.1)	30.1
Ward hall	5865	103.8 (74.3)	30.9	4659	105.0 (74.9)	31.1	1206	99.1 (71.8)	30.2
Other or unknown	5865	19.5 (42.3)	5.8	4659	19.7 (39.6)	5.8	1206	18.6 (51.2)	5.7
Staff area	5865	34.9 (50.2)	10.4	4659	33.4 (49.0)	9.9	1206	40.6 (54.1)	12.4
Transit^b^	5865	17.8 (20.5)	5.3	4659	17.7 (19.2)	5.2	1206	18.1 (24.9)	5.5
Education space	5865	3.3 (15.1)	1.0	4659	4.2 (16.8)	1.2	1206	0.04 (0.5)	0.01
Supply	5865	1.9 (6.6)	0.6	4659	1.9 (6.8)	0.6	1206	1.6 (5.7)	0.5
Family waiting space	5865	3.2 (11.5)	1.0	4659	3.5 (12.0)	1.0	1206	2.4 (9.3)	0.7
Total	5865	335.8 (36.0)	NA	4659	337.8 (33.9)	NA	1206	328.2 (42.3)	NA
12:01 pm to 6:00 pm (afternoon)									
Patient room	4221	40.8 (33.6)	11.6	3361	38.3 (32.2)	10.9	860	50.4 (37.1)	14.3
Physician workroom	4221	131.5 (111.6)	37.5	3361	132.4 (110.0)	37.9	860	128.0 (117.4)	36.2
Ward hall	4221	59.1 (68.0)	16.9	3361	57.9 (67.4)	16.6	860	64.1 (70.0)	18.1
Other or unknown	4221	24.8 (54.5)	7.1	3361	24.7 (54.7)	7.1	860	25.3 (54.0)	7.1
Staff area	4221	50.7 (87.1)	14.5	3361	48.2 (84.5)	13.8	860	60.6 (96.0)	17.1
Transit^b^	4221	21.8 (22.7)	6.2	3361	22.1 (23.9)	6.3	860	20.4 (17.0)	5.8
Education space	4221	17.1 (22.5)	4.9	3361	21.5 (23.3)	6.1	860	0.3 (1.9)	0.1
Supply	4221	2.1 (7.0)	0.6	3361	2.1 (7.4)	0.6	860	1.9 (5.4)	0.5
Family waiting space	4221	2.5 (11.0)	0.7	3361	2.5 (11.1)	0.7	860	2.4 (10.7)	0.7
Total	4221	350.4 (24.1)	NA	3361	349.6 (25.0)	NA	860	353.4 (20.0)	NA
6:01 pm to 12:00 am (evening)									
Patient room	1578	61.7 (41.3)	17.8	1131	61.8 (40.4)	17.9	447	61.2 (43.7)	17.6
Physician workroom	1578	120.9 (104.9)	34.9	1131	121.7 (103.4)	35.2	447	118.9 (109.0)	34.2
Ward hall	1578	78.5 (69.9)	22.7	1131	76.9 (68.2)	22.2	447	82.5 (73.9)	23.8
Other or unknown	1578	21.3 (50.6)	6.1	1131	22.1 (53.0)	6.4	447	19.5 (43.9)	5.6
Staff area	1578	40.5 (56.9)	11.7	1131	40.6 (57.6)	11.7	447	40.4 (55.3)	11.6
Transit^b^	1578	18.6 (17.4)	5.4	1131	18.1 (14.6)	5.2	447	20.0 (22.9)	5.8
Education space	1578	0.1 (2.6)	0.03	1131	0.1 (1.3)	0.03	447	0.2 (4.3)	0.06
Supply	1578	2.9 (9.0)	0.8	1131	2.9 (9.3)	0.8	447	2.9 (8.4)	0.8
Family waiting space	1578	1.7 (9.8)	0.5	1131	1.8 (9.5)	0.5	447	1.5 (10.5)	0.4
Total	1578	346.3 (31.4)	NA	1131	346.0 (31.6)	NA	447	347.2 (31.0)	NA
12:01 am to 6:00 am (night)									
Patient room	2393	48.8 (39.3)	13.8	1699	47.7 (38.6)	13.5	694	51.6 (40.9)	14.6
Physician workroom	2393	110.9 (109.8)	31.3	1699	111.2 (109.9)	31.4	694	110.0 (109.7)	31.0
Ward hall	2393	85.4 (77.5)	24.1	1699	86.3 (77.9)	24.4	694	83.2 (76.4)	23.5
Other or unknown	2393	35.8 (73.0)	10.1	1699	34.5 (70.8)	9.7	694	38.8 (78.0)	11.0
Staff area	2393	53.0 (79.5)	15.0	1699	53.0 (80.1)	15.0	694	52.8 (78.0)	14.9
Transit^b^	2393	16.5 (20.5)	4.7	1699	17.1 (21.3)	4.8	694	14.9 (18.6)	4.2
Education space	2393	0.03 (0.5)	0.01	1699	0.03 (0.5)	0.01	694	0.04 (0.5)	0.01
Supply	2393	2.4 (9.0)	1.0	1699	2.6 (10.0)	0.7	694	2.1 (6.1)	0.6
Family waiting space	2393	1.2 (8.3)	0.7	1699	1.2 (8.7)	0.3	694	1.0 (7.1)	0.3
Total	2393	353.9 (17.7)	NA	1699	353.8 (18.0)	NA	694	354.3 (16.9)	NA

Abbreviation: NA, not applicable.

^a^ For all periods except rounds, person-days with less than 4 hours of location data were excluded because 4 hours was judged to be the minimum amount of time an intern would be in the hospital performing clinical duties. For rounds, there needed to be at least 1 hour of location data for an intern during the rounding times for that intern to be included in analyses for that day.

^b^ Includes elevators, stairways, halls between clinical units, and main hospital hallways; staff area, conference rooms, and break rooms; and supply, medication, and nourishment areas.

### Individual Differences Between Residents in Time at the Bedside

The mean percentage of time at the bedside during a 24-hour period varied among interns from 8.8% to 18.3% (IQR, 11.8%-15.3%; absolute difference between highest and lowest percentages, 9.5%) (Figure and eTable 3 in the Supplement). Model 1, including intern as a random variable, accounted for 8.1% of the overall variance of percentage of time in patient rooms during the 24-hour period, indicating significant differences among interns (χ^2^_1_ = 560.66; *P* < .001) (Table 2). During rounds, interns accounted for 1.4% of the overall variance in time at the bedside, which was also significant (χ^2^_1_ = 118.83; *P* < .001) (Table 3).

**Figure.**
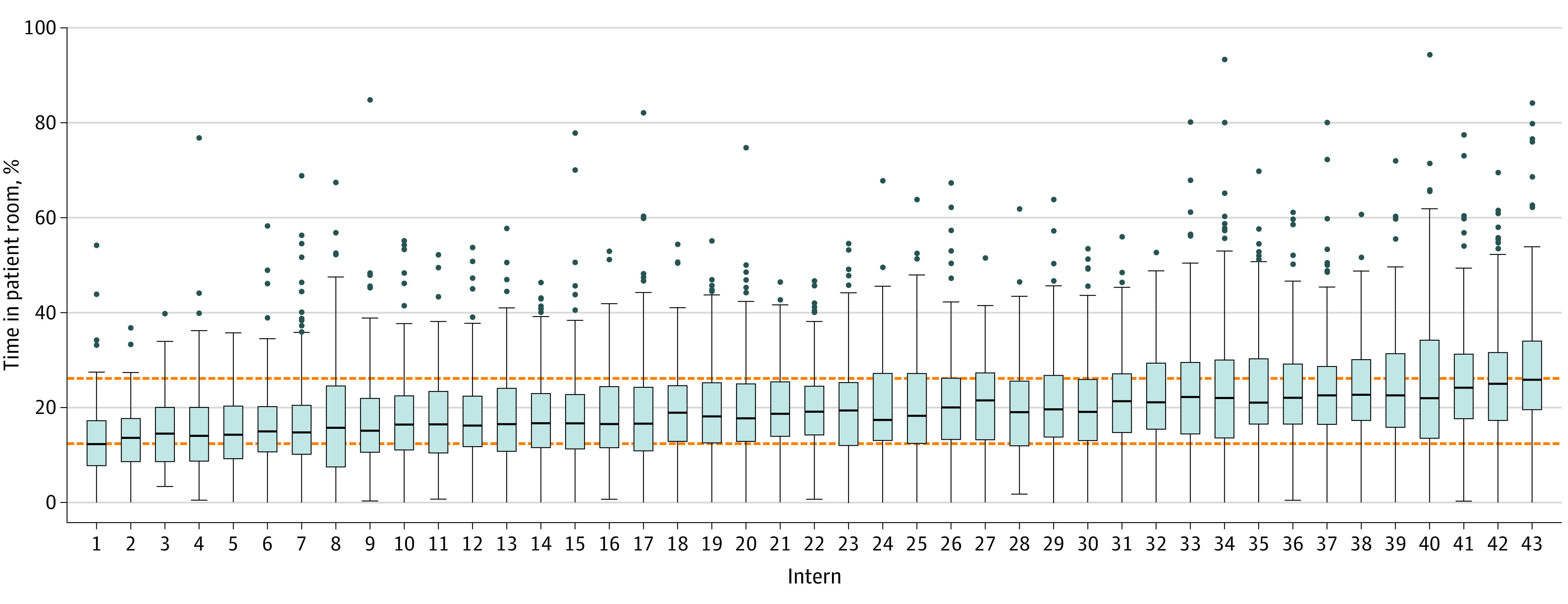
Time in Patient Rooms by Intern Each box plot represents the distribution of the percentage of time at the bedside during a 24-hour period for each intern. Dotted orange lines indicate the IQR for all data. Horizontal lines within the boxes indicate the median time at the bedside. The bottom and top of each box represent the 25th and 75th percentiles, respectively. The lower whisker indicates the smallest value within 1.5 times the IQR below the 25th percentile and the upper whisker indicates the largest value within 1.5 times the IQR above the 75th percentile; blue dots indicate values larger than this threshold.

**Table 2.** Multilevel Modeling Results for Time at Bedside During the 24-Hour Interval

Variable	Model			
	Null	Intern	Intern plus service	Intern plus service plus time
Estimate, β (95% CI) [*P* value]				
Intercept	–0.00 (–0.02 to 0.02) [>.99]	–0.01 (–0.10 to 0.08) [.79]	0.03 (–0.15 to 0.21) [.75]	–0.03 (–0.22 to 0.15) [.72]
Day of year	NA	NA	NA	–0.0004 (–0.0007 to –0.0002) [<.001]
Day of service	NA	NA	NA	0.01 (0.01 to 0.02) [<.001]
Day of week	NA	NA	NA	0.02 (0.01 to 0.03) [<.001]
Random effects				
Within-group variance, σ^2^	NA	0.92	0.90	0.89
Between-group variance, τ_00_				
Intern	NA	0.08	0.08	0.08
Service	NA	NA	0.03	0.03
ICC	NA	0.08	0.11	0.11
No. of interns	NA	43	43	43
No. of services	NA	NA	5	5
No. of observations	7909	7909	7909	7909
*R*^2^/*R*^2^ adjusted	0.000/0.000	0.000/0.081	0.000/0.110	0.005/0.116
Model fit				
AIC	22 448	21 889	21 744	21 709
L ratio (*P* value)	NA	χ^2^_1_ = 560.66 (< .001)	χ^2^_1_ = 146.91 (< .001)	χ^2^_3_ = 40.87 (< .001)

Abbreviations: AIC, Akaike information criterion; ICC, intraclass correlation coefficient; NA, not applicable.

**Table 3.** Multilevel Modeling Results for Time at the Bedside During Rounds

Variable	Model			
	Null	Intern	Intern plus service	Intern plus service plus time
Estimate, β (95% CI) [*P* value]				
Intercept	–0.00 (–0.02 to 0.02) [>.99]	–0.0002 (–0.04 to 0.04) [.93]	–0.01 (–0.40 to 0.37) [.94]	–0.04 (–0.43 to 0.35) [.86]
Day of year	NA	NA	NA	–0.00004 (–0.0002 to 0.0001) [.63]
Day of service	NA	NA	NA	0.01 (0.005 to 0.01) [<.001]
Day of week	NA	NA	NA	–0.01 (–0.02 to –0.001) [.03]
Random effects				
Within-group variance, σ^2^	NA	0.99	0.87	0.87
Between-group variance, τ_00_				
Intern	NA	0.01	0.02	0.02
Service	NA	NA	0.19	0.19
ICC	NA	0.01	0.19	0.19
No. of interns	NA	43	43	43
No. of services	NA	NA	5_e_	5
No. of observations	13 226	13 226	13 226	13 226
*R*^2^/*R*^2^ adjusted	0.000/0.000	0.000/0.014	0.000/0.194	0.002/0.196
Model fit				
AIC	37 537	37 420	35 828	35 804
L ratio (*P* value)	NA	χ^2^_1_ = 118.83 (< .001)	χ^2^_1_ = 1594.20 (< .001)	χ^2^_3_ = 29.50 (< .001)

Abbreviations: AIC, Akaike information criterion; ICC, intraclass correlation coefficient; NA, not applicable.

### Differences in Time at the Bedside Between Services

The mean (SD) percentage of time at the bedside varied by service for the 24-hour period from 11.7% (6.6%) for nononcology subspecialties to 15.4% (5.9%) for oncology, and during rounds from 8.0% (12.4%) for nononcology subspecialties to 26.5% (12.1%) for oncology (Table 4 and eFigure 2 in the Supplement). Model 2, including service as a random variable, demonstrated significantly better fit than model 1 for both the 24-hour interval (χ^2^_1_ = 146.91; *P* < .001) and rounds (χ^2^_1_ = 1594.20; *P* < .001) (Tables 2 and 3). Service accounted for an additional 3.0% of overall variance in time at the bedside during the 24-hour interval and 18.0% during rounds. Random effects for service in the 2 models (Tables 2 and 3 and eFigure 3 in the Supplement) showed small differences in mean time at the bedside across services for the 24-hour period and greater differences during rounds. Compared with the 24-hour period, the mean (SD) percentage of time at the bedside during rounds increased for oncology (from 15.4% [5.9%] to 26.5% [12.1%]), house staff (from 13.1% [6.3%] to 17.5% [15.9%]), and hospitalist services (from 14.3% [9.3%] to 18.6% [17.2%]) and decreased for the ICU (from 14.3% [8.9%] to 9.0% [11.2%]) and nononcology subspecialty services (from 11.7% [6.6%] to 8.0% [12.4%]). There was an increase in the mean (SD) percentage of time spent in ward halls during rounds for oncology (from 18.6% [6.8%] to 50.9% [15.3%]), house staff (from 29.6% [20.5%] to 38.3% [27.7%]), and ICU (from 36.3% [13.8%] to 68.2% [19.4%]) services.

**Table 4.** Time in Locations by Service for 24-Hour and Rounding Periods

Service	Time in location, mean (SD), %					
	Patient room		Ward hall		Physician workroom	
	24-h (n = 7909)	Rounds (n = 13 226)^a^	24-h (n = 7909)	Rounds (n = 13 226)^a^	24-h (n = 7909)	Rounds (n = 13 226)^a^
ICU	14.3 (8.9)	9.0 (11.2)	36.3 (13.8)	68.2 (19.4)	1.2 (4.9)	1.8 (8.7)
House staff	13.1 (6.3)	17.5 (15.9)	29.6 (20.5)	38.3 (27.7)	36.6 (25.5)	21.3 (28.1)
Hospitalist general medicine	14.3 (9.3)	18.6 (17.2)	18.7 (14.6)	19.8 (21.7)	37.5 (25.8)	39.4 (34.9)
Nononcology subspecialty	11.7 (6.6)	8.0 (12.4)	7.8 (8.8)	8.4 (15.3)	56.9 (24.6)	67.4 (37.0)
Oncology	15.4 (5.9)	26.5 (12.1)	18.6 (6.8)	50.9 (15.3)	0.7 (5.1)	0.3 (5.3)

Abbreviation: ICU, intensive care unit.

^a^ Indicates 8:30 am to 11:00 am.

### Changes in Time at the Bedside Over Time 

To test for temporal trends, model 3 included time variables (day of week, day of service rotation, and day of year). For the 24-hour interval, all 3 variables were statistically significant, with day of week (β = 0.02 [95% CI, 0.01-0.03]; *P* < .001) and day of service (β = 0.01 [95% CI, 0.01-0.02]; *P* < .001) having positive associations (interns spent more time at the bedside toward the end of the week and the end of the rotation) and day of year (β = –0.0004 [95% CI, –0.0007 to –0.0002]; *P* < .001) having negative associations (interns spent less time at the bedside toward the end of the academic year) (Table 2). For the rounding interval, day of year was not significant β = –0.00004 [95% CI, –0.0002 to 0.0001]; *P* = .63), day of service was significant and positive (β = 0.01 [95% CI, 0.005-0.01]; *P* < .001), and day of week was significant and negative (β = –0.01 [95% CI, −0.02 to −0.001]; *P* = .03) (Table 3). Model 3 was a significantly better fitting model than model 2 for both the 24-hour interval (χ^2^_3_ = 40.87; *P* < .001) and rounds (χ^2^_3_ = 29.50; *P* < .001) (Tables 2 and 3) but accounted for less than 1% of the overall variance in percentage of time at the bedside in each model, indicating negligible outcomes associated with these temporal variables.

## Discussion

This cross-sectional study used RTLS technology to examine where internal medicine interns spend their time in the hospital. An RTLS is a feasible and scalable tool to quantify the amount of time residents spend with patients, to examine the association between specific clinical rotations and resident time at the bedside, and to explore the effect of specific initiatives on trainee workflow. Our study reported approximately 100 000 hours of resident time in the hospital for a fraction of the cost and personnel of larger, traditional time-motion studies.

We found that interns spend only a small percentage of time in patient rooms, 13.4% overall, which is consistent with prior studies.^5,6^ By some estimates, time at the bedside has decreased by almost half since the 1990s.^25^ A number of factors contribute to less time at the bedside, including operational constraints, duty hour requirements, a focus on patient throughput, and electronic health record workflows.^6,26,27,28^ This shift away from time with patients coincides with a decline in clinical skills and an increase in burnout, particularly among trainees.^8,9,10,14,15,16^ A causal relationship between time at the bedside and these important outcomes, while not established, has become a compelling focus of intervention. There is a growing movement led by organizations such as the Society of Bedside Medicine, the New York Academy of Medicine, and the Accreditation Council for Graduate Medical Education to get residents “back to the bedside” to improve clinical skills and professional fulfillment.^17,29,30,31,32^

We found notable differences in behavior patterns among interns, with some spending nearly twice as much time at the bedside compared with their peers. The absolute difference between the highest and lowest percentages among interns was 9.5%. If we assume an 80-hour work week across a 48-week internship, that difference translates into an additional 365 hours (or 4.5 work weeks) at the bedside for one intern compared with another. The fact that intern status was a significant estimator of time at the bedside suggests that individual trainee characteristics underlie differences in time spent with patients. The fact that time at the bedside did not substantially change during the course of the academic year also suggests that efficiency in resident-specific tasks was not the main reason why interns spent more or less time at the bedside. A better understanding of the reasons for this individual variation in time at the bedside and its association with clinical skills and professional fulfillment could help inform initiatives to improve the graduate medical education experience.

Our study revealed considerable variability at the service level but that little bedside time was incorporated into rounds. Of the 5 clinical services, oncology, house staff, and ICU conducted rounds partially in patient rooms but mostly in ward hallways, and hospitalist and nononcology specialty services spent most rounds in the workroom. This confirms prior studies documenting the shift of morning rounds to hallways and conference rooms.^7,33^

Our findings suggest an opportunity to design initiatives to increase time at the bedside as part of a patient-centered approach to rounds.^34^ Interns on the oncology rotation spent more time in patient rooms on rounds compared with other services. This could reflect a more intentional approach to patient- and family-centered rounds for individuals with cancer, a strategy that improves outcomes in oncology settings.^35^ Interestingly, time spent at the bedside during rounds was the lowest (<10%) for the ICU and nononcology subspecialty services. However, patient-centered multidisciplinary rounds in the ICU can increase rounding efficiency, physician satisfaction, and opportunities for clinical skills teaching.^36^ The RTLS data could help to assess the impact of initiatives designed to increase time at the bedside during rounds in the ICU and other clinical services.

### Limitations

There are limitations to our study. We could not capture data in areas without RTLS receivers (eg, outpatient clinics). We limited our study to internal medicine interns and did not survey nonparticipants to understand why they chose not to participate. These factors limit generalizability to other postgraduate years, nonmedicine specialties, and outpatient clinical work. Although schedules and overall service structures are fairly standardized within each clinical rotation, we did not account for the effect of individual faculty members. Certain periods of time were classified as other or unknown owing to unrealistic times spent in a single location, which could have introduced bias. However, these events represented less than 0.5% of all data and likely did not affect the findings, particularly time spent in patient rooms. We classified rounding time as 8:30 to 11:00 am. Although all services are encouraged to end rounds by 11:00 am, we were not able to track the daily beginning and end times of rounds; some activities may have been misclassified using this approximation. The RTLS technology does not capture activities in a given location. It is possible that interns spent time with patients outside their hospital room or spent most of their time interacting with a computer while in a patient room. Patient rooms were clearly demarcated in the RTLS. However, some areas may have been mislabeled. For example, physician work areas may have been underrepresented in clinical units without standard offices, particularly some ICUs and oncology units. This study was conducted before the COVID-19 pandemic, which had a profound effect on hospital workflow and graduate medical education,^29,37,38^ and may limit generalizability to the current training environment. Installation of an RTLS may be cost prohibitive. However, once installed, the badges are relatively inexpensive. Finally, we do not have data linking RTLS observations with measures of clinical skill and professional fulfillment. Future studies must incorporate RTLS data into a more global assessment of the training environment before this technology can be recommended on a broader scale.^17^

## Conclusions

The findings of this cross-sectional study suggest that an RTLS may be a scalable way to track resident workflows in the hospital and could be used to inform initiatives to improve the residency training environment. Consistent with prior research, we found that interns spend a small proportion of their time in patient rooms. The proportion of time in patient rooms differed by intern, indicating an opportunity for individualized learning interventions. We also found significant differences in rounding time at the bedside by clinical service, suggesting an opportunity to improve the intern and patient experience by focusing on bedside rounding innovations.

## Notes

Supplement. **eMethods.** Reclassification of Badge Hits as Other or Unknown **eTable 1.** Detections of More Than 300 Minutes Recategorized **eTable 2.** Detections From 180 to 300 Minutes Outside of MD Workrooms Recategorized **eTable 3.** Descriptive Statistics for Time in Patient Room for Each Intern **eFigure 1.** Percentage of Time Spent in Locations by Time of Day and Day of Week **eFigure 2.** Distribution of Time at the Bedside, in Ward Halls, and Physician Workrooms Overall and During Usual Morning Rounding Time by Service **eFigure 3.** Random Effects for Service on Percentage of Time at the Bedside During 24-Hour and Rounding Intervals
