# Supplementary material for: Use of a Real-Time Locating System to Assess Internal Medicine Resident Location and Movement in the Hospital
Source: JAMA Netw Open. 2022 Jun 8;5(6):e2215885. doi: 10.1001/jamanetworkopen.2022.15885 (PMC9178434; doi:10.1001/jamanetworkopen.2022.15885)
Supplement: Supplement. — eMethods. Reclassification of Badge Hits as Other or Unknown eTable 1. Detections of More Than 300 Minutes Recategorized eTable 2. Detections From 180 to 300 Minutes Outside of MD Workrooms Recategorized eTable 3. Descriptive Statistics for Time in Patient Room for Each Intern eFigure 1. Percentage of Time Spent in Locations by Time of Day and Day of Week eFigure 2. Distribution of Time at the Bedside, in Ward Halls, and Physician Workrooms Overall and During Usual Morning Rounding Time by Service eFigure 3. Random Effects for Service on Percentage of Time at the Bedside During 24-Hour and Rounding Intervals [file jamanetwopen-e2215885-s001.pdf]

Supplementary Online Content

Rosen MA, Bertram AK, Tung M, Desai SV, Garibaldi BT. Use of a real-time locating system to assess internal medicine resident location and movement in the hospital. *JAMA Netw Open*. 2022;5(6):e2215885. doi:10.1001/jamanetworkopen.2022.15885

eMethods. Reclassification of Badge Hits as Other or Unknown
eTable 1. Detections of More Than 300 Minutes Recategorized
eTable 2. Detections From 180 to 300 Minutes Outside of MD Workrooms Recategorized
eTable 3. Descriptive Statistics for Time in Patient Room for Each Intern
eFigure 1. Percentage of Time Spent in Locations by Time of Day and Day of Week
eFigure 2. Distribution of Time at the Bedside, in Ward Halls, and Physician Workrooms Overall and During Usual Morning Rounding Time by Service
eFigure 3. Random Effects for Service on Percentage of Time at the Bedside During 24-Hour and Rounding Intervals

This supplementary material has been provided by the authors to give readers additional information about their work.

© 2022 Rosen MA et al. *JAMA Network Open*.

eMethods. Reclassification of Badge Hits as Other or Unknown

A total of 9,712 badge detections (0.48% of total detections) were unrealistically long and recategorized as other/unknown. These detections represented 8,645.8 hours, or 9.1% of the total person-hours collected. Three types of detections were recategorized. First, 9,152 detections (92.7% of recoded detections) or 5862.7 hours (64% of the duration of all recoded detections; 6.1% of total time) were longer than 10 minutes in transit areas (i.e. elevators or connecting areas that were not a part of a clinical unit or care delivery area). Second, 185 detections over 300 minutes in duration each were recategorized, representing 1,371.2 hours (16.3% of recoded time; 1.4% of total time). The majority of these detections were in MD Workrooms (672.3 hrs; 49% of recoded time; 0.71% of total time) and Ward Halls (365.3 hrs; 26% or recoded time; 0.38% of total time). A total of 9 detections in Patient Rooms (114.6 hrs; 8.4% of recoded time; 0.12% of total time) were recoded to other/unknown (see **Supplemental Table 1**). Third, 392 detections between 180 and 300 minutes occurring outside of MD Workrooms were recategorized. Of these, 267 were in Ward Halls (960.3 hrs; 68% of recoded time; 1% of total time), 59 in staff or admin areas (209.3 hrs; 14.8% of recoded time; 0.22% of total time), and only 13 (47.0 hrs; 3.3% of recoded time; 0.05% of total time) were in Patient Rooms (see Supplemental Table).

© 2022 Rosen MA et al. *JAMA Network Open*.

**eTable 1.** Detections of More Than 300 Minutes Recategorized				
	**Number of detections**	**Total hours**	**Percentage of recoded time**	**Percentage of total time**
Education	1	16.7	1.20	0.02
Family waiting space	2	17.8	1.30	0.02
MD Workroom	90	672.3	49.00	0.71
Patient room	9	114.6	8.40	0.12
Transit	10	114.6	8.40	0.12
Ward Hall	48	365.3	26.60	0.38
equip/med/nourish	1	9.4	0.70	0.01
staff/admin area	7	60.4	4.40	0.06

© 2022 Rosen MA et al. *JAMA Network Open*.

**eTable 2.** Detections From 180 to 300 Minutes Outside of MD Workrooms Recategorized				
	**Number of detections**	**Total hours**	**Percentage of recoded time**	**Percentage of total time**
Education	10	36.5	2.60	0.04
Family waiting space	9	33.8	2.40	0.04
Patient room	13	47.0	3.30	0.05
Procedure space	4	14.1	1.00	0.01
Transit	27	99.5	7.10	0.10
Ward Hall	267	960.3	68.00	1.01
equip/med/nourish	3	11.3	0.80	0.01
staff/admin area	59	209.3	14.80	0.22

© 2022 Rosen MA et al. *JAMA Network Open*.

eTable 3. Descriptive Statistics for Time in Patient Room for Each Intern

Intern	N	Minutes per 24-hour Period		% time per 24-hour Period		Total Hours Over One Year
		Mean (SD)	*Median (IQR)*	*Mean (SD)*	*Median (IQR)*	
1	233	134.7 (62.5)	130.6 (86.8)	18 (9)	17 (10)	522.9
2	228	121 (59.5)	112.6 (68.2)	16 (7)	15 (9)	459.9
3	234	120.8 (57.1)	117.7 (72.5)	17 (8)	16 (9)	471.1
4	96	118.3 (61.2)	110.3 (67)	15 (7)	14 (9)	189.3
5	168	116.2 (59.7)	107.2 (77.9)	18 (8)	17 (10)	325.4
6	229	115.6 (62.4)	107.1 (71)	16 (8)	15 (10)	441.2
7	187	115.3 (59.3)	105.7 (76.6)	16 (8)	15 (8)	359.4
8	213	112.3 (68.1)	97.1 (76.7)	17 (10)	15 (14)	398.6
9	205	111.1 (53.5)	106.7 (73.1)	15 (6)	14 (8)	379.6
10	180	110.5 (51.2)	105.7 (72)	16 (7)	15 (8)	331.5
11	207	109.9 (57.8)	101.6 (69.4)	16 (8)	14 (9)	379.1
12	199	107.8 (62.3)	92.6 (83.2)	14 (7)	13 (9)	357.7
13	206	107.2 (64.5)	102.4 (79.4)	16 (10)	15 (11)	367.9
14	150	106.8 (60.2)	101.5 (77.4)	16 (9)	15 (10)	266.9
15	98	106.3 (58.4)	93.4 (79.4)	14 (8)	13 (9)	173.6
16	99	102.9 (68.2)	81.6 (82)	14 (8)	12 (9)	169.8
17	218	102.2 (53.1)	88.7 (62.7)	14 (7)	13 (9)	371.3
18	192	100.6 (64.9)	90.2 (72.8)	14 (8)	12 (10)	321.8
19	90	99.7 (59)	91.6 (81.7)	14 (7)	14 (9)	149.6
20	199	99.7 (58.8)	93.1 (77.9)	14 (7)	13 (9)	330.6
21	113	98.8 (53.2)	96.4 (73.1)	13 (7)	13 (8)	186
22	230	97.4 (57.9)	85.5 (79.5)	13 (7)	12 (8)	373.5
23	230	95.9 (57.3)	82.8 (69.2)	13 (7)	12 (8)	367.5
24	82	94.9 (55.8)	88.5 (60.9)	13 (7)	12 (8)	129.7
25	209	94.4 (58.3)	83.3 (65.8)	13 (7)	13 (9)	328.7
26	215	94.2 (48.2)	92.5 (55)	13 (6)	13 (7)	337.6
27	205	89.7 (56.4)	79.1 (71.1)	12 (6)	11 (9)	306.3
28	231	86.7 (56.7)	74.5 (71.2)	12 (7)	11 (9)	333.8
29	228	86 (48)	79 (58.3)	12 (6)	11 (7)	326.7
30	229	85.8 (45)	82 (62.2)	12 (6)	11 (8)	327.4
31	230	84.7 (47.5)	76.1 (62.4)	12 (7)	11 (8)	324.7
32	33	84.5 (48.9)	74.2 (63.1)	10 (6)	9 (6)	46.5
33	232	84.2 (54.5)	76.3 (66)	12 (8)	11 (9)	325.6
34	230	84 (52.8)	70.1 (61.8)	12 (7)	10 (8)	322.1
35	228	83.2 (47.3)	77.7 (56.7)	12 (6)	11 (8)	316.2

© 2022 Rosen MA et al. *JAMA Network Open*.

36	161	81.6 (62.8)	70.5 (83.9)	11 (8)	11 (11)	219.1
37	228	80.2 (46.7)	70.4 (54.1)	11 (6)	10 (6)	304.6
38	177	74.9 (42)	68.6 (49.9)	12 (6)	11 (9)	221
39	53	74.3 (38.4)	70.7 (54.8)	10 (5)	10 (8)	65.6
40	220	72.7 (39.1)	67.3 (46.5)	11 (7)	10 (7)	266.5
41	215	68.4 (40)	62.9 (43.9)	10 (6)	9 (8)	245
42	81	65.9 (31.2)	63.2 (41.1)	10 (5)	10 (7)	89
43	218	63.4 (37.2)	57.6 (48.7)	9 (5)	8 (6)	230.4

N=number of days where badge data was recorded

© 2022 Rosen MA et al. *JAMA Network Open*.

eFigure 1. Percentage of Time Spent in Locations by Time of Day and Day of Week

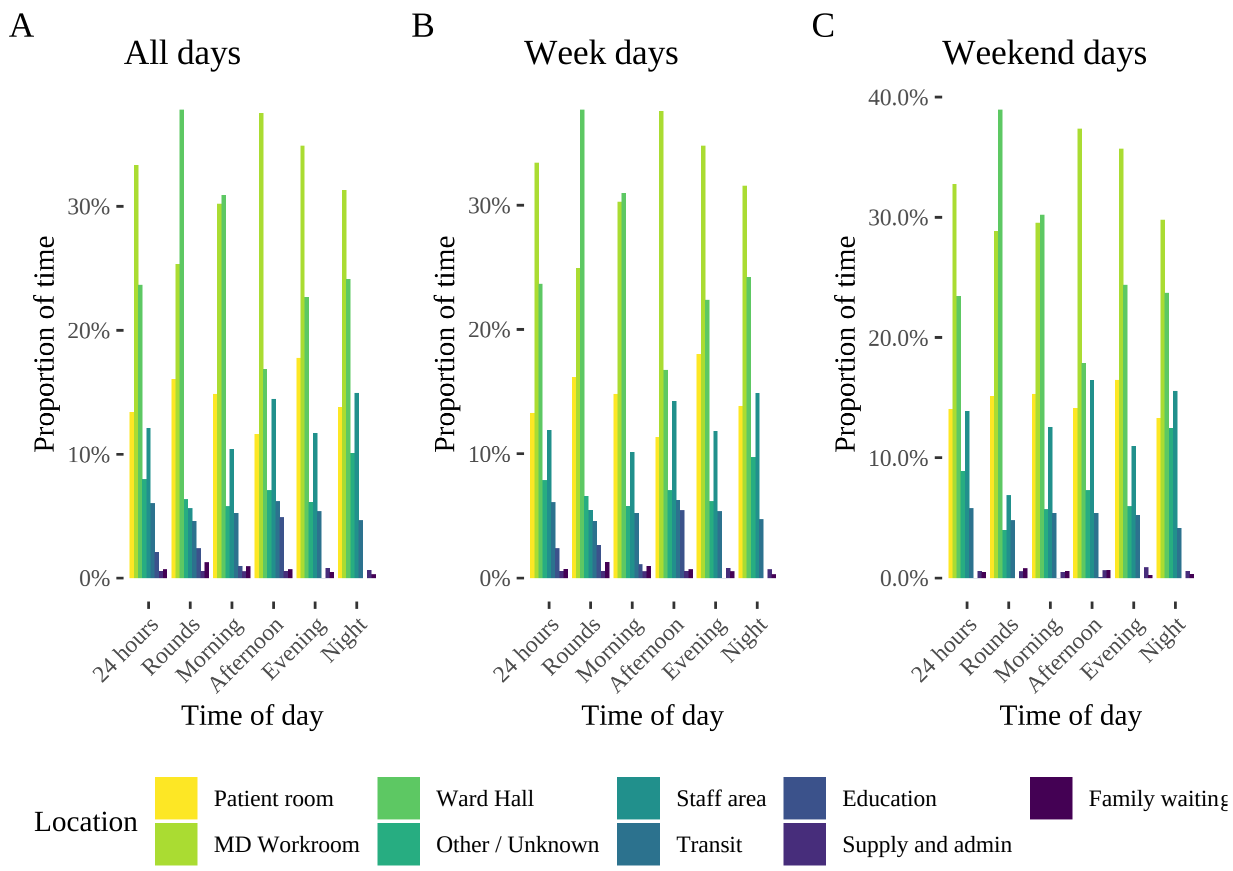

Time ranges:
Morning: 6AM to 12PM
Rounds: 830AM to 11AM
Afternoon: 12PM to 6PM
Evening: 6PM to 12AM
Night: 12AM to 6 AM

© 2022 Rosen MA et al. *JAMA Network Open*.

eFigure 2. Distribution of Time at the Bedside, in Ward Halls, and Physician Workrooms Overall and During Usual Morning Rounding Time by Service

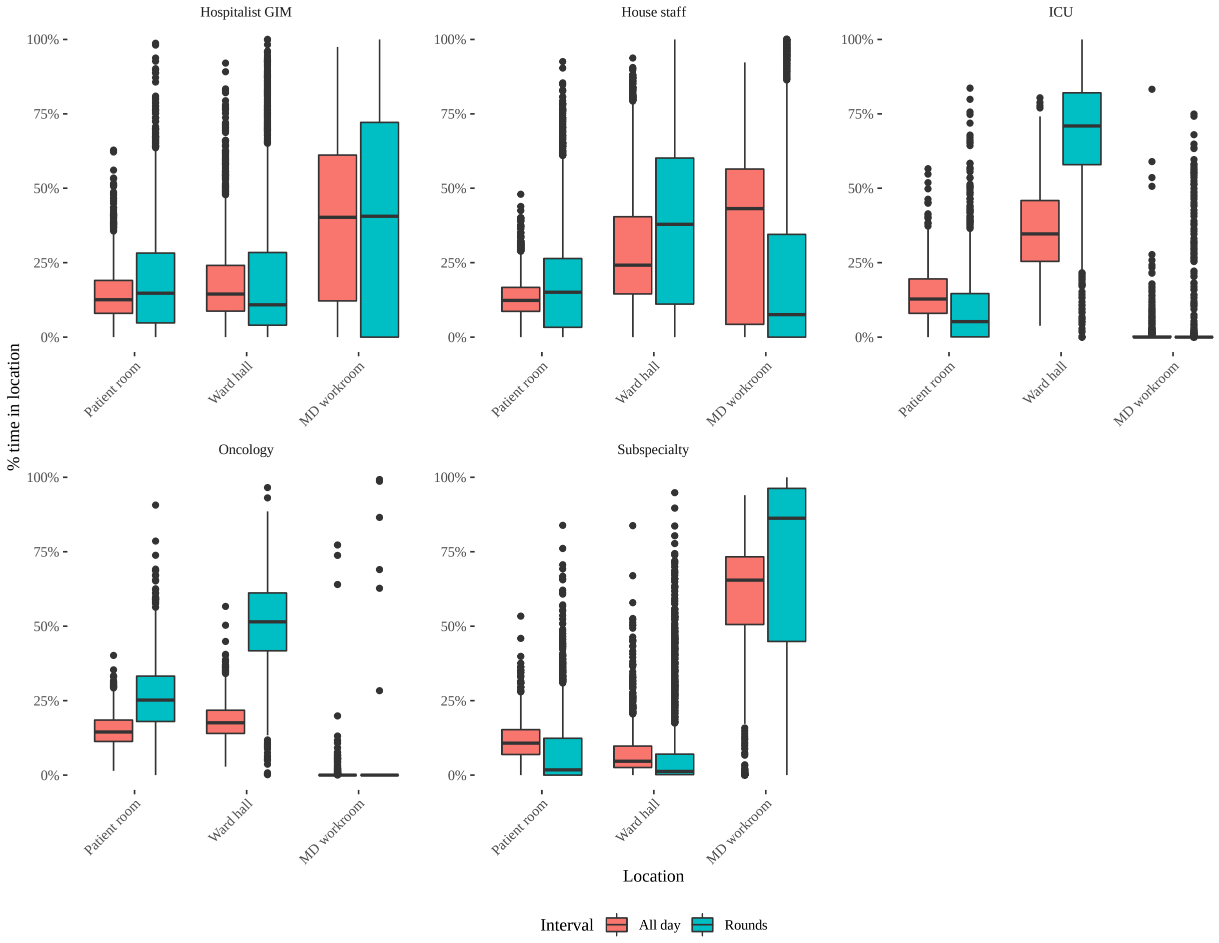

© 2022 Rosen MA et al. *JAMA Network Open*.

eFigure 3. Random Effects for Service on Percentage of Time at the Bedside During 24-Hour and Rounding Intervals

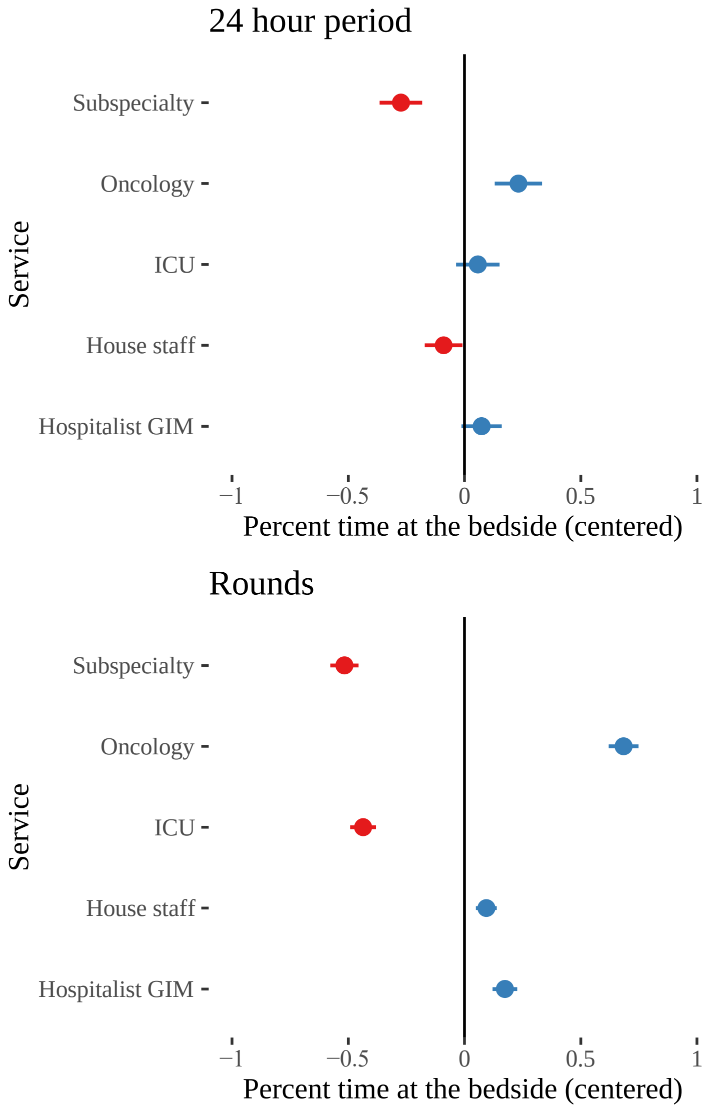

NOTES: Random effects are taken from M_3 models which include all random and fixed effects in this analysis. The x axis is standardized % time at the bedside (0 = mean for the entire sample, 1 unit = one SD) for each time interval. Service mean estimates are shown with 95% CIs.

© 2022 Rosen MA et al. *JAMA Network Open*.
